# Schmerztherapie in der Schwangerschaft: Eine expertInnenbasierte interdisziplinäre Konsensus-Empfehlung

**DOI:** 10.1007/s00482-021-00571-4

**Published:** 2021-07-29

**Authors:** Daniela Marhofer, Wolfgang Jaksch, Thomas Aigmüller, Stefan Jochberger, Berndt Urlesberger, Katharina Pils, Barbara Maier, Rudolf Likar, Beate Kayer, Roswitha Wallner, Petra Fink, Gabriele Grögl

**Affiliations:** 1 Allgemeine Intensivmedizin und Schmerztherapie, Medizinische Universität Wien, Universitätsklinik für Anästhesie, Spitalgasse 23, 1090 Wien, Österreich; 2 Abteilung für Anästhesie, Intensiv- und Schmerzmedizin, Wiener Gesundheitsverbund, Klinik Ottakring, Wien, Österreich; 3 Abteilung für Gynäkologie und Geburtshilfe, Landeskrankenhaus Hochsteiermark – Standort Leoben, Leoben, Österreich; 4 Universitätsklinik für Anästhesie und Intensivmedizin, Medizinische Universität Innsbruck, Innsbruck, Österreich; 5 Universitätsklinik für Kinder- und Jugendheilkunde, Klinische Abteilung für Neonatologie, Medizinische Universität Graz, Graz, Österreich; 6 Institut für Physikalische Medizin und Rehabilitation, Wiener Gesundheitsverbund, Klinik Landstraße, Wien, Österreich; 7 Gynäkologisch-Geburtshilfliche Abteilung, Wiener Gesundheitsverbund, Klinik Ottakring, Wien, Österreich; 8 Abteilung für Anästhesiologie und Intensivmedizin, Klinikum Klagenfurt am Wörthersee, Klagenfurt, Österreich; 9 Fachbereich Hebammen, Fachhochschule Campus Wien, Wien, Österreich; 10 Abteilung für Anästhesiologie und Intensivmedizin, Landesklinikum Amstetten, Amstetten, Österreich; 11 Abteilung für Anästhesie und Operative Intensivmedizin, Wiener Gesundheitsverbund, Klinik Landstraße, Wien, Österreich

**Keywords:** Neuropathischer Schmerz, Migräne, Geburtshilfe, Akuter Schmerz, Chronischer Schmerz, Neuropathic pain, Migraine, Obstetrical, Acute pain, Chronic pain

## Abstract

**Hintergrund:**

Schwangerschaft und Schmerzen unterschiedlicher Herkunft sind eine ungünstige Kombination, die alle BehandlerInnen vor besondere Herausforderungen stellt. Schmerzen beeinflussen die Homöostase des Menschen negativ. Compliance der Patientin und fundiertes Wissen über Fetotoxizität und Teratogenität von Analgetika sind nötig, um die Balance zwischen Therapie der Mutter und Sicherheit des Ungeborenen zu wahren.

**Ziel der Arbeit:**

ExpertInnen verschiedener Disziplinen, die mit der Betreuung von schwangeren Schmerzpatientinnen betraut sind, haben sich zusammengeschlossen, um medikamentöse und nichtmedikamentöse Therapiekonzepte zu erstellen, mit dem Ziel, eine suffiziente Analgesie von schwangeren Schmerzpatientinnen zu ermöglichen.

**Material und Methode:**

Relevante Fragestellungen wurden durch ExpertInnen formuliert und einer Literatursuche unterzogen. Kombiniert mit weiteren nationalen und internationalen Empfehlungen wurden Behandlungskonzepte entwickelt, interdisziplinär diskutiert und im Anschluss Kernaussagen erstellt, die mit Empfehlungsgraden abgestuft wurden.

**Ergebnisse:**

Abhängig vom Trimenon können bei Schmerzen Paracetamol, Ibuprofen, Diclofenac, Metamizol und Opioide wohlüberlegt verabreicht werden, besondere Vorsicht ist bei nichtsteroidalen Antirheumatika (NSAR) im letzten Trimenon geboten. COX-2-Hemmer werden nicht empfohlen. Bei neuropathischen Schmerzen gelten Amitriptylin, Duloxetin, Venlafaxin als sicher. Bei starker Migräne kann Sumatriptan zum Einsatz kommen. Nichtmedikamentöse Therapien wie transkutane elektrische Nervenstimulation (TENS-Therapie), Kinesio-Tapes und Akupunktur gelten als sicher. Lymphdrainagen werden bei Ödemen empfohlen, sofern sie nicht durch eine Präeklampsie bedingt sind.

**Schlussfolgerung:**

Die Therapie sollte mit einer nichtmedikamentösen Behandlung beginnen und eskalierend in einer Kombination aus medikamentösen und nichtmedikamentösen Konzepten enden.

## Abstract (en)

**Background:**

Pregnancy and pain of different origins is an unfavorable combination that presents all practitioners with special challenges. Pain negatively affects the homeostasis of humans. Patient compliance and in-depth knowledge of the fetotoxicity and teratogenicity of the substances are necessary to maintain a balance between therapy for the mother and safety of the unborn child.

**Objectives:**

Experts from various disciplines who are entrusted with the care of pregnant patients with pain have come together to develop drug and nondrug therapy concepts with the aim of providing adequate analgesia for pregnant pain patients.

**Materials and Methods:**

Relevant questions were formulated by experts and subjected to a literature search. Combined with further national and international recommendations, treatment concepts were developed and discussed in an interdisciplinary manner. Core statements were then drawn up and given recommendation grades.

**Results:**

Depending on the trimester, paracetamol, ibuprofen, diclofenac, metamizole, and opioids can be administered carefully in the event of pain; special care is required with nonsteroidal anti-inflammatory drugs (NSAIDs ) in the last trimester. COX‑2 inhibitors are not recommended. For neuropathic pain, amitriptyline, duloxetine, and venlafaxine are considered safe. Non-pharmacological treatment concepts are also available, namely transcutaneous electrical nerve stimulation (TENS therapy), kinesio tapes, and acupuncture. Lymphatic drainage is recommended in cases of edema, if not caused by preeclampsia.

**Conclusions:**

A deliberated concept for pain therapy during pregnancy should be initiated with a non-pharmacological intervention and, if necessary, supplemented with pharmacological agents.

Schmerzen jeglicher Genese stellen schwangere Patientinnen und das Behandlungsteam vor große Herausforderungen. Verschiedenste medikamentöse und nichtmedikamentöse Therapieansätze sind aufgrund mangelnder fachlicher Expertise und Datenlage häufig mit Unsicherheiten hinsichtlich Schädigung des Embryos bzw. Fetus verbunden und werden daher fallweise unterlassen. Aus diesem Grund haben sich ExpertInnen interdisziplinär zusammengeschlossen, um Behandlungsempfehlungen mit dem Ziel zu erstellen, einer schwangeren Patientin mit Schmerzen eine suffiziente Therapie unter Gewährleistung der Sicherheit für das Ungeborene zu ermöglichen.

## Hintergrund

Eine Schwangerschaft kann mit Schmerzen unterschiedlicher Genese und Intensität einhergehen. Schwangerschaftsassoziierte Schmerzen sind am häufigsten durch eine Lockerung des Bindegewebes und des Bandapparates verursacht und werden als muskuloskelettale Schmerzen, seltener, je nach Genese, als abdomineller Schmerz wahrgenommen. Andere Arten von Schmerzen, die nicht mit der Schwangerschaft assoziiert sind, müssen nichtsdestotrotz abgeklärt werden. Bei jährlich fast 85.000 Geburten in Österreich sind Begleiterkrankungen der werdenden Mutter keine Seltenheit. Jede/r BehandlerIn steht vor der entscheidenden Frage, wie gerechtfertigte Bedenken gegen Schmerzmedikamente in Einklang mit einer suffizienten Schmerztherapie einer schwangeren Patientin gebracht werden können. Der Schutz der Schwangerschaft bzw. des Feten hat dabei oberste Priorität. Nichtsdestotrotz muss eine adäquate schmerzmedizinische Versorgung auch in der Gravidität gewährleistet sein. Eine Kombination aus medikamentösen und nichtmedikamentösen Therapieansätzen ist anzustreben.

Schmerzen beeinflussen stressbedingt die Homöostase des menschlichen Organismus und können durch Veränderung der Synthese von pro- und antiinflammatorischen Zytokinen vorzeitige Wehen und eine Frühgeburt auslösen [9]. Die hormonelle Umstellung löst eine Dynamik physiologischer Veränderungen aus, die unmittelbar zu pathologischen Veränderungen führen können, wie zum Beispiel zu Ödembildung oder Überlastung der autochthonen Rückenmuskulatur.

Relevant sind darüber hinaus Pathologien, die unabhängig von der Schwangerschaft auftreten, deren Diagnostik und vor allem Behandlung aber ebenso wichtig sind.

Voraussetzung für den risikoarmen Einsatz eines Analgetikums sind genaue Kenntnisse seines embryotoxischen und fetotoxischen Potenzials. 2 % aller Fehlbildungen haben chemische oder physikalische Ursachen einschließlich Arzneimittel und Drogen [20]. Das Risiko für das Auftreten grobstrukureller Fehlbildungen kann durch die Einnahme bestimmter Medikamente erhöht werden, wobei es, von wenigen Ausnahmen abgesehen, noch immer unter 10 % liegt. Beispiele hierfür sind Kombinationstherapien bestimmter Antikonvulsiva, die Verabreichung von Retinoiden sowie ausgeprägter Alkoholabusus und Polytoxikomanie [20].

Die Empfindlichkeit des Embryos gegenüber toxischen Einflüssen ist in Abhängigkeit von seinem Entwicklungsstadium unterschiedlich stark ausgeprägt. Eine einheitliche Therapiewahl für die Dauer der gesamten Schwangerschaft ist infolgedessen nicht zweckdienlich, hingegen eine adaptierte, vom Trimenon abhängige Empfehlung denkbar.

In der Phase der Organogenese, die etwa 15–60 Tage nach der Befruchtung andauert, ist die Vulnerabilität gegenüber teratogenen Einflüssen am höchsten. In der anschließenden Fetalperiode nimmt sie wieder ab. Toxische Einwirkungen führen dann eher zu Wachstums- und Funktionsstörungen, die Entwicklungsstörungen und Verhaltensauffälligkeiten nach sich ziehen können.

Für ein adäquates Vorgehen soll die Auswahl des Analgetikums basierend auf einer der Schwangerschaftswoche entsprechenden Risikoeinschätzung getroffen werden, die es ermöglicht, aus der in Frage kommenden analgetischen Substanzgruppe ein sicheres Medikament zu wählen, für das die meisten Erfahrungen und geringsten Verdachtsmomente für eine potenzielle Schädigung des Ungeborenen vorliegen.

Um die Art der Schmerzen zu kennen und ihr Ausmaß zu quantifizieren, muss zuerst eine Beurteilung durch die Patientin durchgeführt werden. Dafür steht eine numerische Skala zur Verfügung, auf welcher die subjektiv empfundene Schmerzintensität einer Skala von 0 bis 10 zugeordnet wird, wobei 0 kein Schmerz und 10 der stärkste vorstellbare Schmerz bedeutet. Bei Patientinnen mit Sprachbarrieren empfiehlt sich die Anwendung einer visuellen Skala, mittels derer anhand von Gesichtern ebenso Punkte von 0–10 vergeben werden. Ab einem Ergebnis von größer/gleich 3 in Ruhe und größer/gleich 5 bei Belastung sollte eine adäquate Therapie eingeleitet werden.

Es gibt im deutschsprachigen Raum keinen präzisen wissenschaftlichen Algorithmus, wie Schmerzen einer schwangeren Patientin behandelt werden sollen. Eine wissenschaftliche Auseinandersetzung mit dieser Thematik ist dahingehend schwierig, da die Durchführung von wissenschaftlichen Studien bei Schwangeren heikel ist.

Eine optimale analgetische Versorgung dieses Patientinnenkollektivs stellt ÄrztInnen vor eine immense Herausforderung. Das Internetportal der Berliner Charité, das die Pharmakovigilanz zu einer Vielzahl von Medikamenten für Schwangerschaft und Stillzeit prüft und Informationen zu Verträglichkeiten bietet, ist hilfreich, nichtsdestotrotz ist dies keine praxisrelevante Alternative zu einem evidenten Algorithmus [8].

## Ziel der Arbeit

In diesem Bewusstsein haben ExpertInnen aller in der Geburtshilfe involvierten medizinischen Disziplinen wie Gynäkologie, Anästhesie, Neonatologie, physikalische Medizin sowie Hebammen umfangreiche Therapieempfehlungen entwickelt. Diese sollen einen sicheren Weg im Umgang mit dem Schmerz in der Schwangerschaft ermöglichen.

## Methodik

Österreichische ExpertInnen folgender Gesellschaften bzw. Gruppierungen waren aktiv an der Erstellung der Handlungsempfehlung beteiligt:

- ARGE Geburtshilfe (Arbeitsgruppe Geburtshilfliche Anästhesie der ÖGARI – Österreichische Gesellschaft für Anästhesie, Reanimation und Intensivmedizin)
- ÖSG (Österreichische Schmerzgesellschaft)
- OEGGG (Österreichische Gesellschaft für Gynäkologie und Geburtshilfe)
- BÖPMR (Berufsverband Österreichischer Fachärzte für Physikalische Medizin und Rehabilitation)
- ÖGPMR (Österreichische Gesellschaft für Physikalische Medizin und Rehabilitation)
- ÖGKJ (Österreichische Gesellschaft für Kinder- und Jugendheilkunde)
- ÖHG (Österreichisches Hebammengremium)

Grundlagen der Handlungsempfehlungen waren die Patientinneninformation für Besonderheiten zur Schmerztherapie in der Schwangerschaft und Stillzeit der ÖSG [18] und eine Patientinneninformation für Schmerztherapie in der Schwangerschaft und Stillzeit der Deutschen Schmerzgesellschaft e.V [19]. International hinzugezogen wurden die Patientinneninformation der amerikanischen Gesellschaft für Geburtshilfe und Gynäkologie (American College of Obstetricians and Gynecologists [ACOG]) [17].

Eine kompakte Informationsschrift hat die ÖGARI herausgegeben. Die online Plattform der Berliner Charité zur Embryotoxikologie („embryotox“) und eine Empfehlung zur Schmerztherapie [20] waren ebenfalls dienlich. Die von allen beteiligten Fachgruppen gestellten ExpertInnen definierten Endpunkte, die von allen als relevant angesehenen wurden. Im Anschluss wurde interdisziplinär diskutiert und es wurden abschließend konsensusbasierte Kernaussagen verabschiedet. Jede Expertin/jeder Experte hatte eine gleichrangige Stimme. Als akzeptiert galt eine Aussage, wenn zumindest ein 90%iger Konsensus unter den ExpertInnen bestand. Die Kernaussagen wurden in Empfehlungsgrad A*, A und B gewichtet [21].

- A*: hoher Empfehlungsgrad des Boards, obwohl keine klare Studienlage besteht, es herrscht aber Einigkeit zur Relevanz in der Gruppe
- A: hoher Empfehlungsgrad, der auch mit Literatur zumindest mit einer randomisierten, kontrollierten Studie belegt werden kann
- B: mittelgradiger Empfehlungsgrad, der auch mit Literatur, allerdings nicht mit randomisierten Studien belegt werden kann

## Überblick über Therapieoptionen

### Medikamentöse Therapieoptionen

Folgende Substanzen stehen grundsätzlich zur Verfügung und werden im folgenden Abschnitt hinsichtlich Sicherheit ihres Einsatzes bei Schwangeren beleuchtet.

#### Paracetamol

Aus der aktuellen Datenlage ergibt sich kein Hinweis auf ein erhöhtes Fehlbildungsrisiko [15]. Berichte über einen Zusammenhang zwischen der mütterlichen Paracetamoleinnahme und einer erhöhten Rate von Kryptorchismus haben sich bisher nicht bestätigt. In den letzten Jahren wurden zahlreiche Studien publiziert, die einen möglichen kausalen Zusammenhang zwischen der pränatalen Paracetamolexposition und einem vermehrten Auftreten von asthmatischen Beschwerden, Rhinokonjunktivitis, ekzematösen Hautveränderungen sowie erhöhten IgE-Werten im Kindesalter darstellen [3, 5]. Die Ergebnisse dieser Publikationen sind widersprüchlich, eine fundierte Erklärung für diesen postulierten Zusammenhang steht noch aus.

Eine Kausalität zwischen der pränatalen Einnahme von Paracetamol und dem Auftreten von autistischen Symptomen speziell bei männlichen Nachkommen sowie der Entwicklung eines Aufmerksamkeitsdefizit-Hyperaktivitäts-Syndroms und von Sprachentwicklungsstörungen bei weiblichen Nachkommen ist nicht belegbar [2, 20].

#### Nichtsteroidale Antirheumatika (NSAR)

Die Einnahme von NSAR im ersten Trimenon wird wiederholt mit einem erhöhten Abortrisiko in Verbindung gebracht. Die derzeit vorliegende Studienlage erlaubt keine endgültige Bewertung eines möglichen Zusammenhangs. Zur Verzerrung des Studienergebnisses führt oftmals die Verabreichung eines NSAR zur Behandlung abdomineller Schmerzen, die einem Abort vorangehen. Daraus lässt sich aber keine Korrelation zwischen der NSAR-Verabreichung und einem erhöhten Abortrisiko ableiten [12].

Es liegen bisher unbestätigte Hinweise für ein gehäuftes Auftreten von Kryptorchismus vor. Aufgrund der Prostaglandinsynthesehemmung kann die Anwendung von NSAR im dritten Trimenon zu einem frühzeitigen Verschluss bzw. einer Verengung des Ductus arteriosus Botalli (DA) führen. Je reifer der Fetus ist, desto größer ist die Wahrscheinlichkeit eines vorzeitigen Verschlusses unter NSAR-Therapie. Daraus ergibt sich die Empfehlung, NSAR im dritten Trimenon nur in absoluten Ausnahmefällen und so kurz wie möglich anzuwenden (s. Tab. 1). Eine Doppler-sonografische Kontrolle des fetalen Kreislaufs ist bei Verabreichung von NSAR im dritten Trimenon unumgänglich und soll regelmäßig durchgeführt werden.

Eine Einnahme im letzten Schwangerschaftsdrittel kann Nierenfunktionsstörungen mit Entwicklung eines Oligohydramnions hervorrufen bzw. bis in die Neonatalphase nachwirkend zu Oligurie/Anurie führen [7]. Ursächlich für die gestörte Nierenfunktion wird eine renale Minderperfusion verbunden mit einem Anstieg von Vasopressin angesehen [28].

Die bei Neugeborenen zu beobachtende nekrotisierende Enterokolitis nach pränataler NSAR-Exposition wird derzeit auf eine mögliche mesenteriale Minderperfusion beim Feten zurückgeführt [1]. In der Literatur wird vielfach über die Problematik einer NSAR-Einnahme während der Schwangerschaft diskutiert. Das teratogene Risiko von NSAR scheint nach derzeitiger Datenlage vernachlässigbar zu sein. Hinweise auf ein erhöhtes Risiko für das Auftreten von An- und Mikrophthalmie sowie von Neuralrohrdefekten, Spaltbildungen und Pulmonalklappenstenosen haben sich bisher nicht bestätigt [13].

#### Coxibe

Anwendungsbeobachtungen sind nicht in ausreichendem Maße vorhanden, ebenso wenig wie klinische Erfahrungen mit Coxiben, da diese Substanzen noch nicht lange genug im Umlauf sind. Aufgrund der Prostaglandinsynthesehemmung sind in der zweiten Schwangerschaftshälfte fetotoxische Effekte wie unter NSAR-Einnahme zu erwarten [30].

#### Acetylsalicylsäure (ASS)

Eine Low-Dose-Behandlung mit ASS bis maximal 300 mg/Tag zur Prophylaxe und Therapie der Präeklampsie ist während der gesamten Schwangerschaft unbedenklich. Die analgetische, antipyretische und antiphlogistische Wirkung erfolgt über eine Hemmung der Prostaglandinsynthese und setzt erst bei Einzeldosen von 500 mg ein. Im dritten Trimenon kann es unter analgetischer ASS-Therapie zu einer Verengung oder einem frühzeitigen Verschluss des DA kommen. Ein erhöhtes Risiko für das Auftreten postnataler intrakranieller Blutungen findet sich primär bei Frühgeborenen, wenn ASS in analgetischer Dosierung unmittelbar präpartal verabreicht wird. Der peripartale mütterliche Blutverlust kann nach ASS-Einnahme erhöht sein [7].

Aus der derzeitigen Datenlage ergibt sich kein Hinweis auf ein relevantes teratogenes Risiko. Zusammenhänge von ASS-Einnahme und einem gehäuften Auftreten von Gastrochisis, Nierenfehlbildungen und Kryptorchismus haben sich bisher nicht bestätigt [20].

#### Metamizol

Aus wissenschaftlichen Daten ergeben sich keine Hinweise auf ein teratogenes Risiko.

Der jeweils nur in einer Studie angeführte Zusammenhang zwischen der mütterlichen Einnahme von Metamizol und dem Auftreten von Wilms-Tumoren sowie von frühkindlichen Leukämien hat sich in weiteren Studien und in Verlaufsbeobachtungen nicht bestätigt [4, 11, 20].

Aufgrund der Prostaglandinsynthesehemmung kann die Anwendung im dritten Trimenon zu einem frühzeitigen Verschluss oder zu einer Verengung des DA führen. Daher gelten die gleichen strengen Indikations- und Behandlungsrichtlinien wie bei NSAR (s. Tab. 1).

Unter den Nicht-Opioid-Analgetika weist Metamizol als einziges Medikament eine spasmolytische Wirkung auf.

#### Opioide

Für Opioide gibt es derzeit keine Hinweise auf ein erhöhtes Fehlbildungsrisiko. Das in einigen Studien angeführte Risiko für kardiale Fehlbildungen hat sich nicht bestätigt. Opioide, sowohl schwach wirksame wie zu Beispiel Tramadol als auch stark wirksame wie zum Beispiel Buprenorphin, können daher in allen Phasen der Schwangerschaft eingesetzt werden [31] (Tab. 2). Unzureichend untersuchte Opioide wie Tapentadol sollten gemieden werden [20].

**Table 1:** 

	1. Trimenon	2. Trimenon	3. Trimenon
1. Wahl	Paracetamol (TMD: 4 g) und Ibuprofen (TMD: 2400 mg)	Paracetamol (TMD: 4 g) und Ibuprofen (TMD: 2400 mg)	Paracetamol (TMD: 4 g)
2. Wahl	Diclofenac (TMD: 150 mg)	Diclofenac (TMD: 150 mg)	–
3. Wahl	Metamizol (TMD oral: 4 g, i.v: 5 g)	Metamizol (TMD oral: 4 g, i.v: 5 g)	–

**Table 2:** 

1. Wahl (starke Schmerzen)	Tramadol (plus Antiemetikum Metoclopramid) (TMD: 400 mg)
2. Wahl (sehr starke Schmerzen)	z. B. Buprenorphin, Hydromorphon, Oxycodon, Fentanyl

Bei einer Dauertherapie oder Substitutionsbehandlung sind Entzugssymptome beim Neugeborenen zu erwarten, die eine intensive postnatale Überwachung des Kindes erfordern [16, 23]. Einige Studien zeigen für Buprenorphin eine milder verlaufende Entzugssymptomatik beim Neugeborenen. Naloxon darf bei entsprechender Indikation in allen Phasen der Gravidität verabreicht werden.

Werden Opioide eingesetzt, muss vorübergehend mit einer eingeschränkten Variabilität der kindlichen Herzfrequenz und mit verminderten Kindsbewegungen gerechnet werden. Die Wirkung auf den Fetus bzw. auf das Neugeborene ist von Zeitpunkt und Dauer der mütterlichen Exposition, der plazentaren Transferrate, der kindlichen Metabolisierungs- und Ausscheidungsrate und dem Vorhandensein aktiver Metaboliten abhängig.

Pethidin ist sehr gut plazentagängig und erreicht im Feten höhere Spiegel als in der Mutter. Angesichts der unreifen und daher verminderten Metabolisierungsrate beim Feten und Neugeborenen ist die Halbwertszeit für Pethidin und seinen neurotoxisch wirkenden Metaboliten Norpethidin um ein Vielfaches verlängert. Daraus resultieren neonatale Nebenwirkungen wie erniedrigte Apgar-Werte, Atemdepression, respiratorische Azidose und kindliche Krampfanfälle. Sie treten gehäuft nach wiederholter Applikation von Pethidin während des Geburtsvorgangs auf.

Zur Therapie bzw. Prophylaxe von opioidinduzierter Übelkeit und Erbrechen gilt Metoclopramid als vertretbar [20].

#### Migränetherapie und Triptane

Bei leichter Migräne kann Paracetamol während der gesamten Schwangerschaft verabreicht werden, NSAR und Metamizol können im ersten und zweiten Trimenon angewendet werden (Tab. 3; [10]). Zur antiemetischen Therapie kann Metoclopramid während der gesamten Gravidität eingesetzt werden.

**Table 3:** 

	1. Trimenon	2. Trimenon	3. Trimenon
Leichte Migräne	Paracetamol (TMD: 4 g), NSAR Ibuprofen (TMD: 2400 mg) und Diclofenac (TMD: 150 mg), Metamizol (TMD: oral 4 g, i.v. 5 g)	Paracetamol (TMD: 4 g), NSAR Ibuprofen (TMD: 2400 mg) und Diclofenac (TMD: 150 mg), Metamizol (TMD: oral 4 g, i.v. 5 g)	Paracetamol (TMD: 4 g)
Schwere Migräne	Sumatriptan (TMD: 300 mg)	Sumatriptan (TMD: 300 mg)	Sumatriptan (TMD: 300 mg)
Reserve	Andere Triptane	Andere Triptane	Andere Triptane
Prophylaxe	Amitriptylin (TMD: 150 mg) und Metoprolol (TMD: 200 mg)	Amitriptylin (TMD: 150 mg) und Metoprolol (TMD: 200 mg)	Amitriptylin (TMD: 150 mg) und Metoprolol (TMD: 200 mg)

Bei schwerer Migräne können Triptane verabreicht werden, Sumatriptan sollte der Vorzug gegeben werden, da dazu die umfangreichste Datenlage besteht (Tab. 3; [10]). Bisher finden sich keine Hinweise für ein teratogenes Risiko der in der Akuttherapie zum Einsatz kommenden Triptane [20, 22, 25, 26]. Bei unzureichender Wirkung oder Unverträglichkeit können Zolmitriptan und Rizatriptan eingesetzt werden.

Zur Migränetherapie sollte in der Gravidität primär immer ein Behandlungsversuch mit nichtmedikamentösen Therapieverfahren wie z. B. Entspannungstechniken, ätherischen Ölen, Akupunktur oder Akupressur erfolgen.

Für die Migräneprophylaxe können Metoprolol und Amitriptylin zum Einsatz kommen [20]. Die Verabreichung von Antikonvulsiva ist aufgrund des teratogenen Risikos kontraindiziert.

#### Lokalanästhetika

Lokalanästhetika können während der gesamten Schwangerschaft für Regionalanästhesie, invasive schmerztherapeutische Verfahren und Infiltrationen angewendet werden. Der Anwendung des Ultraschalls sollte der Vorzug gegeben werden, da die Erfolgsrate von Blockaden durch direkte Visualisierung von anatomischen Strukturen, Nerven eingeschlossen, mittels Ultraschall hoch ist.

Negative Auswirkungen auf die Neurophysiologie des Neugeborenen oder teratogene Schäden sind bisher nicht beobachtet worden. Ist eine Anästhesie notwendig, sollte, wenn möglich, immer auf ein regionales Verfahren zurückgegriffen werden, um eine systemische Gabe von Medikamenten zu vermeiden und das erhöhte Aspirationsrisiko im Zuge einer Vollnarkose während einer Schwangerschaft zu reduzieren.

#### Therapie neuropathischer Schmerzen

Neuropathische Schmerzen, die bereits vor Eintritt der Schwangerschaft bestanden haben, müssen ungeachtet der Schwangerschaft weiter behandelt werden. Die Therapie sollte an die Gravidität adaptiert weitergeführt werden. Daraus resultierende mögliche Therapieänderungen sollten im Konsens mit der bereits behandelnden Fachdisziplin erfolgen.

### Co-Analgetika zur Therapie neuropathischer Schmerzen

#### Antikonvulsiva

Antikonvulsiva sollten in der Schwangerschaft zur Behandlung neuropathischer Schmerzen nicht oder nur in Ausnahmefällen bei fehlenden Alternativen verabreicht werden. Weder für Gabapentin noch für Pregabalin kann nach derzeitiger Datenlage ein teratogenes Risiko mit Sicherheit ausgeschlossen werden. Für Carbamazepin ist ein teratogenes Risiko, das vor allem das Auftreten von Neuralrohrdefekten triggert, nachgewiesen. Beim Einsatz von Antikonvulsiva im ersten Trimenon sollte eine spezifische Ultraschalluntersuchung des Embryos angeboten werden.

Die beste Datenlage findet sich für Lamotrigin, das jedoch in der Therapie des neuropathischen Schmerzes nur eine untergeordnete Rolle spielt. Bis zu einer Tageshöchstdosis von 300 mg Lamotrigin scheint kein teratogenes Risiko vorzuliegen. Höhere Tagesdosen weisen eine etwas erhöhte Fehlbildungsrate auf. Jedoch ist Lamotrigin aufgrund der schlechteren Wirksamkeit bei neuropathischen Schmerzen nur als Reservemedikation anzusehen, eine Tagesdosis von 300 mg darf verabreicht werden (Tab. 4).

**Table 4:** 

1. Wahl	Amitriptylin (TMD: 150 mg)
2. Wahl	Duloxetin (TMD: 120 mg)
3. Wahl	Venlafaxin (TMD: 225 mg)
Reservemedikation	Lamotrigin (empfohlene TMD: 300 mg)

Da die Lamotrigin-Clearance in der Schwangerschaft stark ansteigen kann, müssen die Plasmaspiegel engmaschig kontrolliert werden.

#### Antidepressiva

Für trizyklische Antidepressiva liegen keine Hinweise auf ein teratogenes Risiko vor. Die beste Datenlage weist Amitriptylin auf (Tab. 4). Für die selektiven Serotonin- und Noradrenalin-Wiederaufnahmehemmer Duloxetin und Venlafaxin zeigen die bisher ausgewerteten Schwangerschaftsverläufe kein erhöhtes Fehlbildungsrisiko (Tab. 4; [20, 24]). Bei kontinuierlicher Verabreichung bis zur Geburt können Anpassungsstörungen beim Neugeborenen auftreten. Eine Entbindung sollte daher in einer Klinik mit angeschlossener Neonatologie erfolgen [29].

### Nichtmedikamentöse Therapieoptionen

Schwangerschaftsbedingte Veränderungen stellen die häufigsten Ursachen für Schmerzen im Bereich des Rückens und Beckens dar. Die Förderung eines biopsychosozialen Krankheitsverständnisses mit kontinuierlicher Aufklärung sowie Motivation der Betroffenen zu einer gesunden Lebensführung, die regelmäßige körperliche Bewegung beinhaltet, ist ein erster wesentlicher Schritt. Einfache Übungen und gezielte Bewegungsprogramme können in den Alltag integriert werden, um Beschwerden zu lindern. Pilates und Yoga werden speziell für Frauen in der Schwangerschaft angeboten und unterstützen einerseits die Kräftigung der Muskulatur, andererseits wirken sie der schwangerschaftsassoziierten Fehlhaltung (Hyperlordose der LWS) entgegen. Durch gezielte Übungen kann der Rektusdiastase und den konsekutiven Beschwerden entgegengewirkt werden [14].

Beckenbodentraining und eine Stabilisierung bzw. Kräftigung der paraspinalen Muskulatur sind nicht nur in der Schwangerschaft, sondern auch als Vorbereitung für die Geburt wichtig. Bewegungstherapie darf ab dem zweiten Trimenon nicht in Bauchlage durchgeführt werden.

Ein entsprechendes Gewichtsmanagement ist ebenso wichtig wie die richtige Bewegung beim Heben und Tragen bzw. die Haltungskorrektur.

Wärmeanwendungen schwächen Missempfindungen bei Schwangerschaftswehen und der Dehnung von Mutterbändern ab. Schonung und Ruhigstellung werden bei Schmerzen empfohlen. Isometrische Übungen sind daher wichtig, um die Muskulatur zu erhalten. Entspannungsverfahren wie Atemtechniken, progressive Muskelrelaxation nach Jacobson und autogenes Training sind wirksam bei psychischen und physischen Störungen wie Angst, Schlafstörungen und Schmerzerlebnissen aus dem gesamten Muskelbereich.

Werden physikalische Therapien angewendet, gilt TENS Elektrotherapie (Transkutane elektrische Nervenstimulation) als sicher [27]. Dabei handelt es sich um eine nebenwirkungsfreie Therapie bei Schmerzen im thorakolumbalen Übergang, die mehrmals pro Woche als Heimtherapie durchgeführt werden kann.

Für andere Formen der Elektrotherapie gibt es keine Evidenz. Hochfrequenztherapie ist während der gesamten Schwangerschaft kontraindiziert.

Kinesio-Tapes, die im Lendenwirbelbereich paravertebral und über dem Iliosakralgelenk oder zur Unterstützung des Bauchs geklebt werden, zeigen vor allem im dritten Trimenon eine gute Evidenz und sind risikolos für Mutter und Kind.

Therapeutischer Ultraschall in einem Frequenzbereich von 800–1000 Hz kann an Armen und Beinen angewandt werden, ist aber im Bereich des Bauchs und des unteren Rückens kontraindiziert.

Soft-Lasertherapie kann ohne Risiko über Schmerz- oder Akupunkturpunkte angewandt werden.

Akupunktur ist in der Schwangerschaft beliebt und wird von schwangeren Frauen als nichtpharmakologische Methode gerne in Anspruch genommen. Einsatz findet sie bei jeglichen schwangerschaftsbedingten Beschwerden, beispielsweise Kreuzschmerzen. Studienergebnisse weisen darauf hin, dass es bei regelmäßiger Anwendung zu einer Verbesserung kommen kann [6]. Lymphdrainagen sind bei Ödemen empfehlenswert, in erster Linie bei assoziierten Nervenkompressionssyndromen. Bei Eiweißmangelödemen bringen sie keinen Benefit. Bei Ödemen im Rahmen der Präeklampsie sind Lymphdrainagen nur in Ausnahmefällen indiziert.

## Fazit für die Praxis

Kernaussage Paracetamol

- Paracetamol wird nach wie vor als Analgetikum der *ersten Wahl* angesehen. ÄrztInnen sollen während der gesamten Schwangerschaft darauf zurückgreifen (Empfehlungsgrad A).

Kernaussage NSAR

- Gut erprobte NSAR, wie Ibuprofen und Diclofenac, sollen in den ersten beiden Trimena bevorzugt eingesetzt werden, wobei Ibuprofen neben Paracetamol als Analgetikum der *ersten Wahl *angesehen wird (Empfehlungsgrad A*).
- Im letzten Trimenon sollen NSAR nur unter absolut strengster Indikationsstellung und kürzestmöglich zum Einsatz kommen (Empfehlungsgrad A*).
- Ist der Einsatz von NSAR im dritten Trimenon unverzichtbar, sollen sowohl fetaler Kreislauf Doppler-sonografisch als auch Fruchtwassermenge sonografisch regelmäßig kontrolliert werden (Empfehlungsgrad B).

Kernaussage Coxibe

- Die Anwendung selektiver COX-2-Inhibitoren in der Schwangerschaft wird derzeit aufgrund der mangelhaften Datenlage *nicht empfohlen* (Empfehlungsgrad A).

Kernaussage ASS

- In analgetischer Dosierung (≥ 500 mg) kann ASS in den ersten beiden Trimena zur Anwendung kommen (Empfehlungsgrad A*).
- Im letzten Trimenon soll ASS in analgetischer Dosierung (≥ 500 mg) nur unter strengster Indikationsstellung zum Einsatz kommen (Empfehlungsgrad A*).
- Die bis zur Vollendung der 37. Schwangerschaftswoche empfohlene Präeklampsie Prophylaxe mit ASS bis zu 300 mg/d ist von dieser Empfehlung nicht betroffen, ebenso nicht betroffen ist die Kardioprotektion bis 100 mg/d (Empfehlungsgrad A).

Kernaussage Metamizol

- Im dritten Trimenon soll die Verabreichung von Metamizol nur unter absolut strengster Indikationsstellung und so kurz wie möglich erfolgen.
- Wie bei NSAR sollen fetaler Kreislauf und Fruchtwassermenge regelmäßig ultrasonografisch kontrolliert werden (Empfehlungsgrad B).

Kernaussage Opioide

- Prinzipiell können Opioide während der gesamten Schwangerschaft zum Einsatz kommen (Empfehlungsgrad A*).
- Die Anwendung von Tapentadol wird derzeit aufgrund der mangelhaften Datenlage nicht empfohlen (Empfehlungsgrad A*).
- Nach einer Opioid-Langzeittherapie der Mutter muss mit Entzugssymptomen des Neugeborenen gerechnet werden. Deshalb soll peripartal eine neonatologische Versorgung sichergestellt sein (Empfehlungsgrad A).
- Als Antiemetikum zur Therapie und Prophylaxe gilt Metoclopramid als akzeptabel. (Empfehlungsgrad A).

Kernaussage Migräne

- Zur Therapie leichter Migräneattacken darf Paracetamol während der gesamten Schwangerschaft verabreicht werden (Empfehlungsgrad A).
- Für NSAR, Metamizol und Metoclopramid gelten die gleichen Richtlinien wie bereits oben erwähnt.
- Für die Therapie schwerer Migräneattacken soll primär Sumatriptan verordnet werden (Empfehlungsgrad A).
- Bei unzureichender Wirkung oder Unverträglichkeit können auch andere Triptane eingesetzt werden (Empfehlungsgrad B).
- Zur Migräneprophylaxe können Amitriptylin und Metoprolol während der gesamten Schwangerschaft verabreicht werden (Empfehlungsgrad A*).

Kernaussage Lokalanästhetika

- Lokalanästhetika können während der gesamten Schwangerschaft für Regionalanästhesieverfahren, schmerztherapeutische Verfahren und Infiltrationen zur Anwendung kommen (Empfehlungsgrad A).
- In der Schwangerschaft soll, wenn immer möglich, Regionalanästhesie bevorzugt werden (Empfehlungsgrad A).

Kernaussage neuropathische Schmerzen

- Das trizyklische Antidepressivum Amitriptylin soll zur Therapie von neuropathischen Schmerzen verabreicht werden (Empfehlungsgrad A).
- Selektive Serotonin- und Noradrenalin-Wiederaufnahmehemmer wie Duloxetin und Venlafaxin können in der Gravidität verabreicht werden (Empfehlungsgrad A*).
- Antikonvulsiva sollten zur Therapie von neuropathischen Schmerzen nur als Ultima Ratio angewendet werden (Empfehlungsgrad A*).

Kernaussage nichtmedikamentöse Therapie

- Bewegungstherapie, TENS-Elektrotherapie, Kinesio-Tapes, Akupunktur und Soft-Lasertherapie gelten als sichere Therapiemethoden und können in der gesamten Schwangerschaft angewendet werden (Empfehlungsgrad B).
